# The American Pocket Medical Dictionary

**Published:** 1904-01

**Authors:** 


The American Pocket Medical Dictionary. Edited by W. A. Newman Dorland, M. D., Assistant Obstetrician to the Hospital of the University of Pennsylvania. Containing the pronunciation and definition of the principal words used in medicine and kindred sciences, with 566 pages and 64 extensive tables. Philadelphia, New York, London: W. B. Saunders & Company. 1903. (Flexible leather, with gold edges, $1.00 net; with thumb index, $1.25 net.)

The fourth edition of this convenient book is issued in an enlarged and revised form. Many words have been added, and altogether it is now as practical and useful a small dictionary as one could wish for everyday use. It is a handy bit of literature to have on the desk for hurried reference work, and is as near perfect as such a dictionary could be made.

